# Elevated temperature and drought stress significantly affect fruit quality and activity of anthocyanin-related enzymes in jujube (*Ziziphus jujuba* Mill. cv. ‘Lingwuchangzao’)

**DOI:** 10.1371/journal.pone.0241491

**Published:** 2020-11-05

**Authors:** Wenqian Jiang, Na Li, Dapeng Zhang, Lyndel Meinhardt, Bing Cao, Yuanjing Li, Lihua Song

**Affiliations:** 1 School of Agriculture, Ningxia University, Yinchuan, Ningxia, China; 2 USDA-ARS, NEA, BARC, SPCL, Beltsville, MD, United States of America; Huazhong Agriculture University, CHINA

## Abstract

The quality attributes of jujube fruit can be directly and indirectly affected by abiotic stresses associated with climate change. Increased temperature and drought are among the most important factors challenging sustainable jujube production in the temperate semi-arid region in northwest China. The main objective of the present study was to understand the effects of elevated air temperature and drought stress on sugar and acid accumulation and coloration of jujube fruits. The content of soluble sugar, organic acid and pigments of traditional jujube "Linwuchangzao" under different atmospheric temperatures and drought stresses were analyzed during three different fruit ripening stages. The elevated temperature (1.5–2.5° C than normal temperature) significantly increased the fruit sugar content, sugar-acid ratio, anthocyanins, flavonoids and carotenoids content. Under the drought stress where the soil moisture was 30% -50% of the field capacity, sugar content, anthocyanin, flavonoid and carotenoid content of the fruit were significantly reduced at the same temperature, but the chlorophyll and organic acid content increased. No significant interaction of Temperature x Drought was observed for all the analyzed quality parameters. The current results showed that the fruit quality of jujube variety "Lingwuchangzao" could be improved when the atmospheric temperature increases by 2° C in this region. However, drought stress had a negative impact on the fruit's sugar-acid ratio and pigment content. The present results also showed that the synthesis and accumulation of anthocyanins in jujube fruit were positively correlated with sugar content and related enzyme activities, especially Phenylalanine Ammonia-lyase (PAL) activity. This study, therefore, provides novel information for understanding the influence of growth environment on the quality properties of jujube fruits. This knowledge will help develop appropriate crop management practices for jujube production in arid and semi-arid areas in northwest China.

## Introduction

Increased temperature and drought are among the most important factors challenging sustainable agricultural production worldwide [1–3]. The world average temperature is likely to increase by 1.5°C between 2030 and 2052 [4] and 2.3 ± 0.3°C by 2070 [5, 6]. Along with these increased temperatures, it is also predicted that in the late 21st century there will be changes to precipitation patterns in various regions and an increase in intensity and/or duration of drought on a regional to global scale [7]. If these changes in are expected to occur over the coming decades, then it’s essential to understand their potential impacts on food production, in terms of quantity and quality, and develop adaptation strategies to offset these impacts [8, 9]. While the impact analysis and adaptation studies have been increasingly carried out for major field crops, a large knowledge gap remains for specialty perennial crops, which makes an important contribution to our global diet [10].

Jujube (*Ziziphus jujuba* Mill.), also known as Chinese Date, is one of the earliest domesticated fruit trees in the world [11, 12]. This fruit crop is native to China but is becoming increasingly popular globally for its outstanding adaptability to marginal land and a broad range of climate conditions. It is an ideal crop in arid and semi-arid areas of temperate and subtropical regions, where other fruit trees do not grow well. This feature makes jujube trees an important economic crop and has made important contributions to poverty reduction and afforestation in China [11, 13]. The crop has been widely planted in about 50 countries in Asia, Europe, Africa, America and Oceania [13–15].

Jujube fruits can be consumed as fresh dates and processed dried dates. The two types of consumption usually require different varieties, cultivation management, harvest time and post-harvest management. Fresh dates are usually harvested before they are fully ripe, in order to have the best quality, flavor and a good balance of sugar and acid content. Jujube fruit undergo significant texture and color changes during fruit development and postharvest ripening [16] The flavor and external attractiveness (color) are the most important quality attributes that determine consumer recognition and commercial value [13, 17]. High-quality fruit typically have a deep red color, high sugar content and a balanced sugar-to-acid ratio [18, 19]. The color change of jujube fruit mainly depends on the content and proportion of chlorophyll, carotenoids, flavonoid, and anthocyanins in the fruit peel [20]. The type and content of pigment determine the color of jujube fruit. The green and yellow colors of the fruit (epidermis) are derived from chlorophyll and carotenoids, while the red and reddish-purple mainly determined by the content and proportion of phenolic acid, flavonoids, flavanols and anthocyanins, which are associated with antioxidant activity and the medicinal properties of jujube fruit [16, 21–23].

Environmental factors significantly affected fruit quality of horticultural crops [19, 24–27]. It is reported that high temperature significantly affects the accumulation of sugar content and organic acid content degradation of fruit [18, 23, 28]. High temperature was reported to adversely affect fruit coloration by inhibiting the expression of anthocyanin activators and related structural genes and/or enhancing that of repressors [29–31]. Higher temperatures in melons increased the accumulation of sucrose in the fruit [32]. Drought stress stimulates secondary metabolism, thereby having an influence on fruit flavor and coloration [33]. It was reported that moderate water deficit could positively affect the soluble sugar content, the sugar/acid ratio of the jujube [34, 35]. The soluble solid content, acid content and sugar/acid ratio content of pear-jujube fruit [34], apricot [36], peach [37] were all enhanced as a result of deficit irrigation. Increased anthocyanin accumulation was induced by the up-regulation of genes involved in drought stress [38–40]. Higher anthocyanins content was also reported in other fruit crops, such as apricots [36, 41], pomegranates [42], apples [43, 44], and strawberries [45–47] with the deficit irrigation treatment. However, negative effects of severe water limitations on the content of anthocyanins have also been reported, because drought stress can reduce photosynthesis resulting in poor fruit color development [48].

So far little information is available on the environmental impact of jujube fruit quality attributes. Moreover, most of the published studies have been focused on a single stress factor and concentrated on a single stage of development. It is unclear how the effect of increasing temperature on jujube color interacts with soil moisture. In semi-arid temperate regions, such as many jujube-producing regions in northwestern China, jujube growers must consider the effects of both water stress and rising temperatures due to global climate change. Different abiotic stresses may affect the primary and secondary metabolism of jujube in different ways, thereby changing their quality attributes, including the flavor, texture and color of the fruit [13, 49]. This knowledge will play a key role in enabling us to predict the potential impact of climate change on jujube production in Northwest China.

The current experiment was designed to examine more precisely the effect of increased atmospheric temperature and drought stress on the fruit quality attributes, including soluble sugar, organic acid, sugar/acid ratio, pigments and anthocyanin content, as well as the activities of key enzymes related to anthocyanin synthesis. The two-factor experiment covered the whole fruit developmental stages to gain insight on the changes of fruit quality parameters, in relation to the temperature and drought treatments. Our study provides scientific knowledge for developing appropriate crop management practices for jujube production in the semiarid regions in northwest China, under the background of global climate change.

## Materials and methods

### Plant materials and growth condition

**Fig 1:**
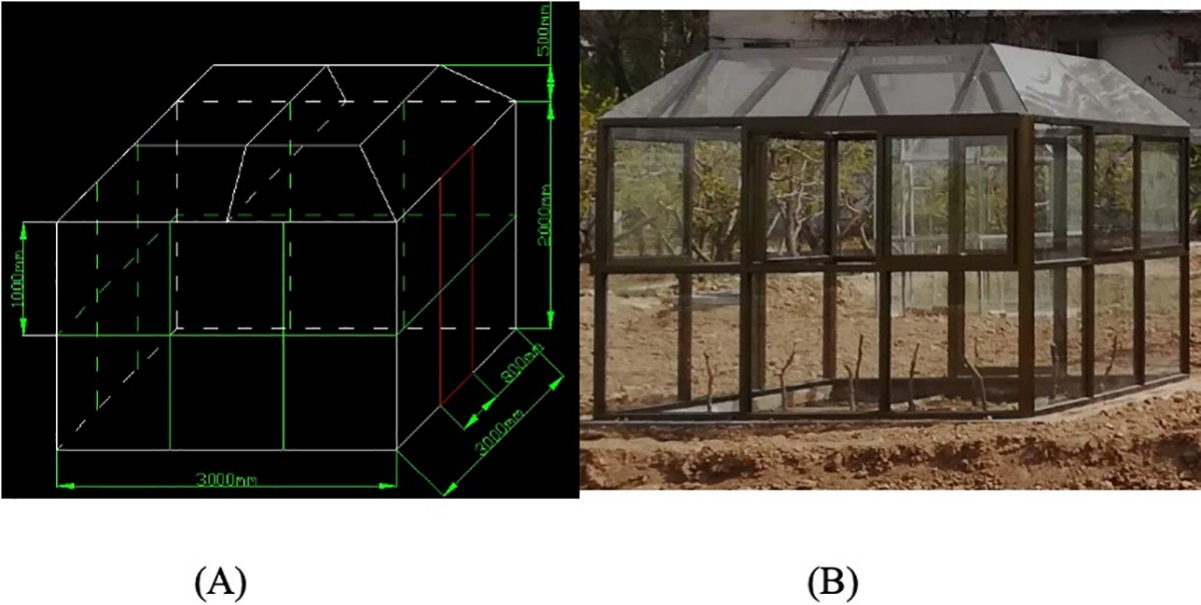
(A and B) The detailed design of open top chambers.

The jujube fruits (*Ziziphus jujuba* Mill. cv ‘Lingwuchangzao’) were obtained from the experimental farm of Ningxia University (Yinchuan, Ningxia, China, 38°47′07″N, 106°04′00″E). Yinchuan has a temperate arid climate and the average annual temperature in this region is about 8.5° C and the average annual rainfall is 180 to 200 mm [50]. The experiments were conducted in open top chambers (OTCs) (Fig 1A and 1B) with two factors treatment, air temperature and drought stress. The orientation of all plots was in a north-south configuration. The average tree height was 1.5 meters and the plants were spaced in a 1x1 arrangement.

### The treatment of elevated air temperature and drought stress

The treatment of drought stress included three levels (D1, 70–75% of field capacity; D2, 50–55% of field capacity and D3, 30–35% of field capacity), whereas the treatment of air temperature included two levels (T1, natural air temperature; T2, elevated air temperature = T1+(2 (±0.5)°C). A detailed description of the two treatments is presented in Table 1.

**Table 1:** Treatment of atmospheric temperature and soil moisture and the experimental design.

	Normal temperature	Elevated temperature
	(T1)	(T2 = T1+ (2.0°C±0.5°C))
Normal soil moisture(D1)	D1T1	D1T2
Moderate drought (D2)	D2T1	D2T2
Severe drought (D3)	D3T1	D3T2

**Fig 2:**
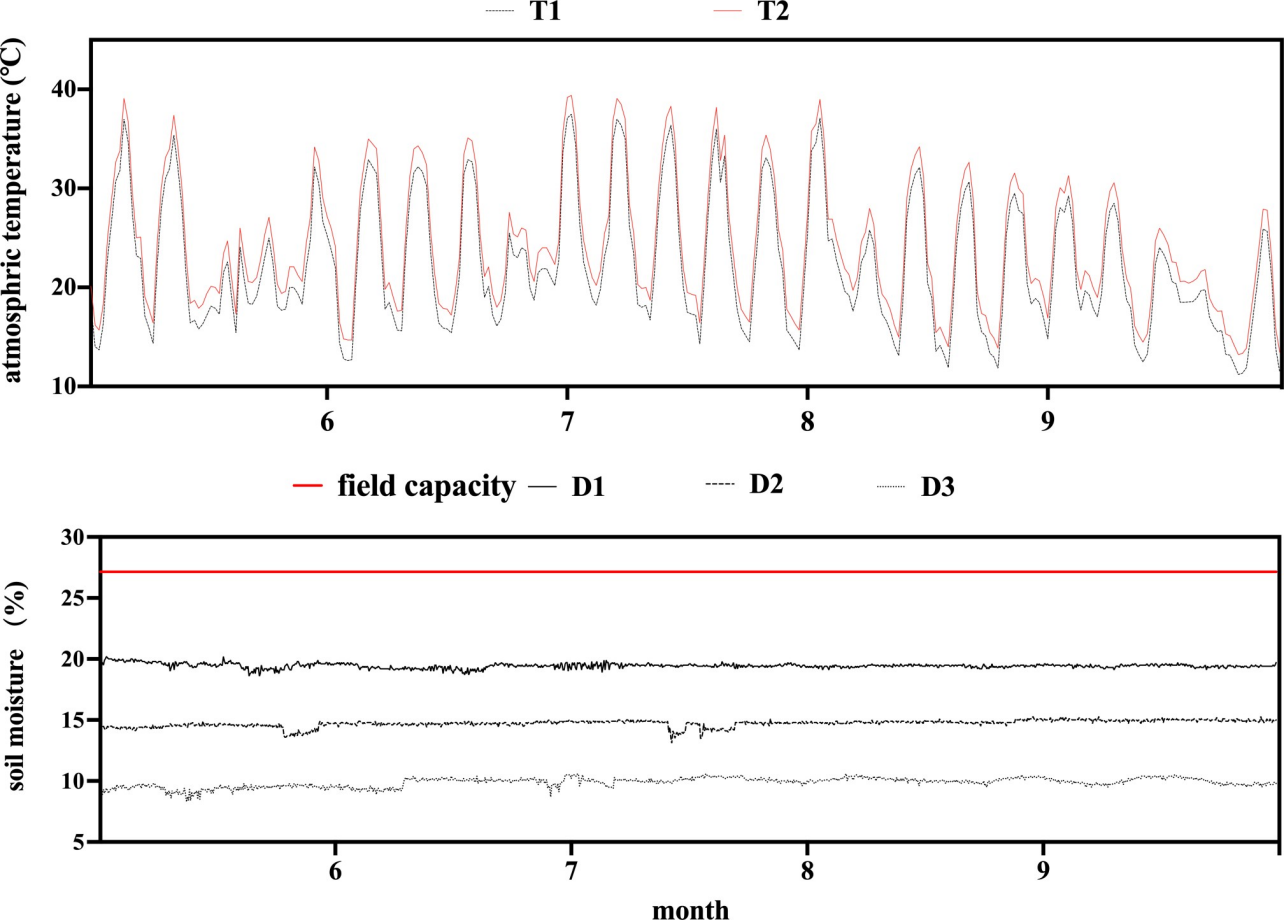
Trend of air temperature and soil moisture in the OTC.

Fifty-four jujube trees were planted in 6 OTCs that simulated the environment with elevated temperature. A solar automatic irrigation system was used to regulate and control soil moisture in each OTC. Air temperature and soil moisture in the OTCs were monitored to meet the experimental requirements using a multi-channel wireless data acquisition equipment (ZWSN-C-A). The data were collected and uploaded every 30 minutes. Data of 6 days in each month (5th, 10th, 15th, 20th, 25th and 30th of each month) were used to plot the temperature and soil moisture changes in the OTCs (Fig 2). The soil moisture of each experiment in the OTCs were within the established parameters (with a measured water holding capacity of 27% in the field), and the temperature difference between the natural temperature and elevated temperature was 1.9–2.5°C. It showed that both the simulated elevation in temperature and soil moistures meet the requirements of the experiment (Fig 2).

### Plant sample preparation

For each tree, a sample of 15 to 20 jujube fruits were collected from marked fruit clusters during the fruit white ripening stage (S1, 08/26/2018), the coloring stage (S2, 09/20/2018), and the complete ripening stage (S3, 10/01/2018) to ensure that the collected fruits had the same flowering time and fruit development (jujube has an infinite inflorescence). Once the fruits were taken, the samples were transferred to the laboratory immediately in a cooler with ice packs and stored in the refrigerator at 4°C for the determination of soluble sugar, organic acid, pigment content and -80°C for the determination of the activity of enzymes related to anthocyanin synthesis.

### Analysis of soluble sugar, organic acid, pigments and enzyme activities

#### Soluble sugars

Soluble sugars were determined following the method described by the Plant Physiology and Biochemistry Experiment Guide [51]. One gram of fruit pericarp tissue with 15 mL distilled water in a test tube was boiled for 20 min in a boiling water bath (HWS-26, Yiheng Corp, Shanghai, China), and was filtered into a 100 mL volumetric flask after cooling to room temperature. The residue was thoroughly washed with distilled water and volume adjusted. The extracts were boiled again in a water bath for 10 min and then cooled to room temperature. Three replicates were used to represent each sample. The absorbance of the supernatant was measured at 620 nm using a visible spectrophotometer (723N, Yidian Analytical Instrument Corp, Shanghai, China) with mix distilled water and anthrone reagent as the control. The recorded absorbance was used to compute the content of chlorophyll and carotenoid. Optical density and glucose content (μg) were used to develop a standard curve. Determination of sample liquid was done by taking 1.0 mL of the extract of the sample to be tested and adding 5 mL of anthrone reagent, and determine the optical density in the same way as above, repeated three times. Soluble sugar content (%) = amount of sugar found from the standard curve (μg) × volume of extraction solution (ml) × dilution factor (100) / [volume of sample solution measurement (mL) × weight of sample (g) × 10^6^] ×100%. Averages were calculated for the three repetition measurements after the content calculations.

#### Organic acid

Organic acid content was determined using the method of Zou [51]. Ten grams of fruit pericarp tissue was ground in 30 mL of distilled water. Then it as introduced into a 50 mL Erlenmeyer flask. After 30 min in a 80°C water bath the samples were cooled and filtered, take 10 mL of the filtrate to the 50 mL Erlenmeyer flasks and 3 drops of phenolphthalein was add by the dropper, then the samples were titrated with 0.1mol / L NaOH to a pinkish color, that did not fade for 30s. Organic acid content (%) = (A × 0.1 × k × c) / (W × D). A: The amount of NaOH consumed. K: 0.067. C: The total amount of dilution. W: Sample weight. D: Determination of sampling volume. Average the three repetitions measurements after the content calculation.

#### Chlorophyll and carotenoids content

The total chlorophyll and carotenoids were determined according to the method of Zou [51]. Fruit skin of approximately 1 mm thick was hand peeled using a fruit peeler. The peels were immediately frozen and ground into powder in liquid nitrogen. 1 gram of the powder was ground in 10 ml of 80% acetone precooled to 4°C to form a homogenate and then the residue was thoroughly washed with 80% cold acetone until the residue is colorless. The mixture was stored in the dark at 4°C for 24 hours to extract, then centrifuged at 12000 rpm for 20 minutes. The absorbance of the supernatant was measured at 470 nm, 646 nm and 663nm by an ultraviolet spectrophotometer (1260, Agilent Technology, Palo Alto, CA, USA), and 80% acetone was used as a control. The recorded absorbance is used to calculate the content of chlorophyll and carotenoids following the formula: Chlorophyll content (mg / g FW) = (17.32 A645 + 7.18 A663) × V / 1000 m; Carotenoid content (mg / g FW) = (1000 A470-563.3 A663-2623.41 A645) × V / 1000 m, where V is the volume of the extract (mL); m is the weight of the peel (g). Averages were calculated for the three repetition measurements after the content calculations.

#### Anthocyanins and flavonoids

The anthocyanin content was determined according to the method of Pirie and Wang [52, 53]. Fifteen fruits were hand-peeled and ground into powder with liquid nitrogen. 2.5 gram of powder were taken and added to 25 mL of 1% HCl-methanol to a 50mL Erlenmeyer flask and extracted in the dark at 4° C for 24 hours. The extract was centrifuged at 12000 rpm for 20 minutes, and then the supernatant was measured at 325 nm, 530 nm, and 665 nm by an ultraviolet spectrophotometer (1260, Agilent Technology, Palo Alto, CA, USA) with 1% HCl-methanol as a control. Anthocyanin content (mg·g^-1^ FW) = △A * 0.005 * 1000 * 445.2 / (30200 * 2.5), △A = A530-A657; 0.025 is the volume of the extract (L); 445.2 is anthocyanin 3- Molar mass of galactosidase molecule; 30200 is the molar specific absorption coefficient of cyanidin 3-galactosidin; 2.5 is the peel weight (g). The content of flavonoids is directly expressed as U2 = OD325nm·g-1FW. Averages were calculated for the three repetition measurements after the content calculations.

#### Enzyme activity (PAL, CHI, DFR and UFGT)

The one mm thick fruit skins were hand-peeled using a fruit peeler. Add an appropriate amount of liquid nitrogen in a mortar to grind the peels of 15 fruits into powder. A sub-sample of 0.5 g powdered peels was taken and extracted in a 5 ml flask with extract solution [0.05 mol·L-1Na_2_HPO_4_ / KH PO_4_ (pH 7.0), 0.05 mol·L^-1^ ascorbic acid, 0.018 mol·L^-1^ mercaptoethanol]. The slurry was centrifuged at 15000g for 20 min at 4° C, and the supernatant was used as the crude enzyme extract for the determination of PAL and CHI enzyme activities. One gram of sample was grinded with liquid nitrogen and 5 ml of pre-cooled acetone was added and mixed. After centrifuging, the supernatant was discarded and the pellet was extracted again with 4ml of acetone, followed by precipitation. The supernatant was used as crude extract of DFR and UFGT enzymes. TCHI, PAL, DFR, UFGT ELISA detection kits were used to determine the enzyme activity in the enzyme label analyzer (Rayto RT-6100, China) according to manufacturer’s recommendations.

### Statistical analysis

The data were analyzed using a two-way analysis of variance (ANOVA), with both temperature and drought stress as fixed factors. If the ANOVA results of drought × temperature was significant, Tukey’s post-hoc test was used for multiple comparisons. All data were determined in triplicate and expressed as the mean ± standard error (SE). Analyses were conducted using SPSS 23.0 (SPSS, IBM Corporation, Armonk, NY, USA). The data were plotted using Graphpad Prism 8.0 software (Graphpad, University of California, Harvey Motulsky, CA, USA).

## Results

### Total sugar, organic acid content and sugar-acid ratio

**Fig 3:**
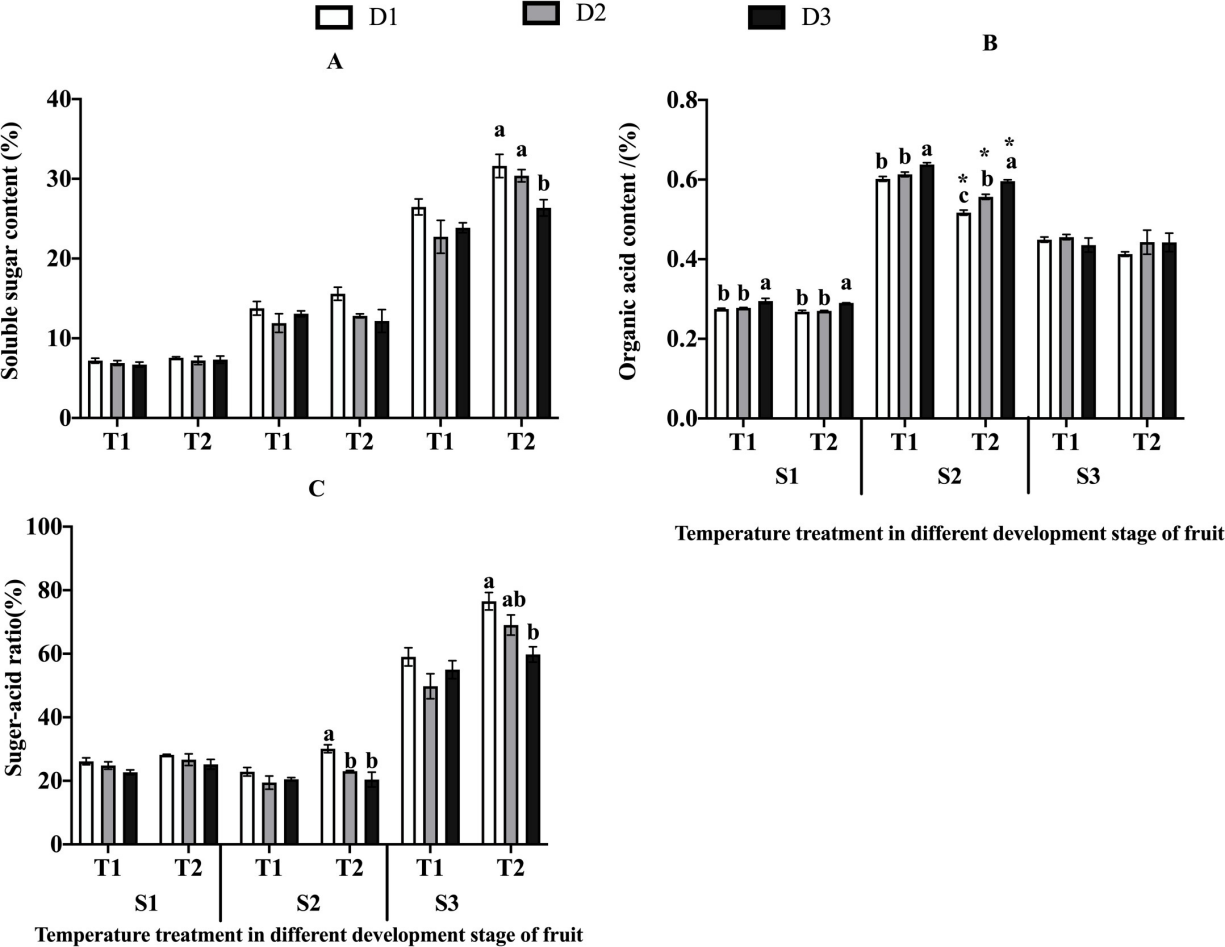
Texture indices in jujubes. Values are means ± SE (n≥3). (A) The content of soluble sugar in jujubes. (B) The content of organic acid in jujubes. (C) The sugar-acid ratio. Different letters in bars indicant significant differences between means for the drought stress on the same temperature treatments at the same period using Duncan’s test (*p* <0.05). * indicant elevated temperature has a significant effect on the same drought stress levels (*p* <0.05).

There was no significant difference in the soluble sugar content among the different temperature and drought stress in the stages of white ripening (S1) and fruit coloration (S2), but both elevated temperature and drought had significant (P<0.05) and highly significant (P<0.01) effects on soluble sugar content in S3 (Table 2). The Temperature x Drought interaction was not significant. The soluble sugar content increased under elevated temperature and decreased with the intensification of drought stress during all three development stages (S1, S2, S3) (Fig 3A). Specially, the degree of decrease resulting from drought stress was not consistent under different temperature. During S3, the soluble sugar content decreased more significantly with the D3 treatment under T2 condition than that under T1 condition, the soluble sugar content decreased significantly 16.61% compared with D1 (Fig 3A).

**Table 2:** Results of two-way ANOVA (F-value) on effects of elevated temperature and drought stress on the fruit quality.

		Total soluble sugar content	Organic acid content	Sugar-acid ratio
S1	Temperature	2.136	5.61*	4.529
	Drought	0.599	24.734**	3.45
	Temperature × Drought	0.099	0.088	0.042
S2	Temperature	0.67	178.017**	8.626*
	Drought	3.823	53.356**	9.671**
	Temperature × Drought	1.148	7.606**	3.027
S3	Temperature	24.89**	0.941	31.336**
	Drought	5.05*	0.531	6.591*
	Temperature × Drought	2.122	0.73	3.391

**: *P<*0.01

*: *P<*0.05. The same below.

For organic acid content, there were significant (P<0.05) and highly significant (P<0.01) difference among the different temperature and drought stresses for the stages of white ripening (S1) and fruit coloration (S2). And the interaction (Temp. x Drought) was significant for organic acid in S2. (Table 2). The organic acid content of fruits decreased under elevated temperature and increased when the drought stress increased from S1 to S3 (Fig 3B). During S2, the organic acid content increased more significantly (P<0.05) with the intensification of drought stress under T2 than that under T1, increasing by 7.68%, and 15.34% compared with D1. And the organic acid content decreased more in T2 than that T1 under different drought levels (D1, D2 and D3), decreasing by 14.10%, 9.22% and 6.56%, respectively (Fig 3B). At S1, no interaction in the organic acid content was observed between elevated temperature and drought stress (Table 2).

For sugar-acid ratio, there were significant (P<0.05) and highly significant (P<0.01) difference among the different temperature and drought stresses in the stages of fruit coloration (S2) and complete ripening (S3), but the elevated temperature and drought stress showed no significant effect in the stage of white ripening (S1) (Table 2). The sugar-acid ratio of fruit increased under elevated temperature and decreased when the drought stress increased across S1 to S3 (Fig 3C). The decrease of sugar-acid ratio by drought stress under T2 condition was more significant than that under the T1 condition in S2 and S3. However, the Temp. x Drought was not significant for sugar-acid ratio in S2 and S3 (Table 2).

The changes in the content of soluble sugar, organic acid and sugar-acid ratio in different treatment combinations at different developmental stages is also presented in Fig 3A–3C. The highest soluble sugar content was observed in S3 (T2D1), while the lowest content was in S1 (T1D3). For the content of organic acids, the highest value was in S2 (T1D3), while the lowest value was observed in S1 (T2D1). The highest sugar-acid ratio was observed in S3 (T2D1), while the lowest sugar-acid ratio was observed in S2 (T1D2) (Fig 3A–3C).

### Pigment contents

**Fig 4:**
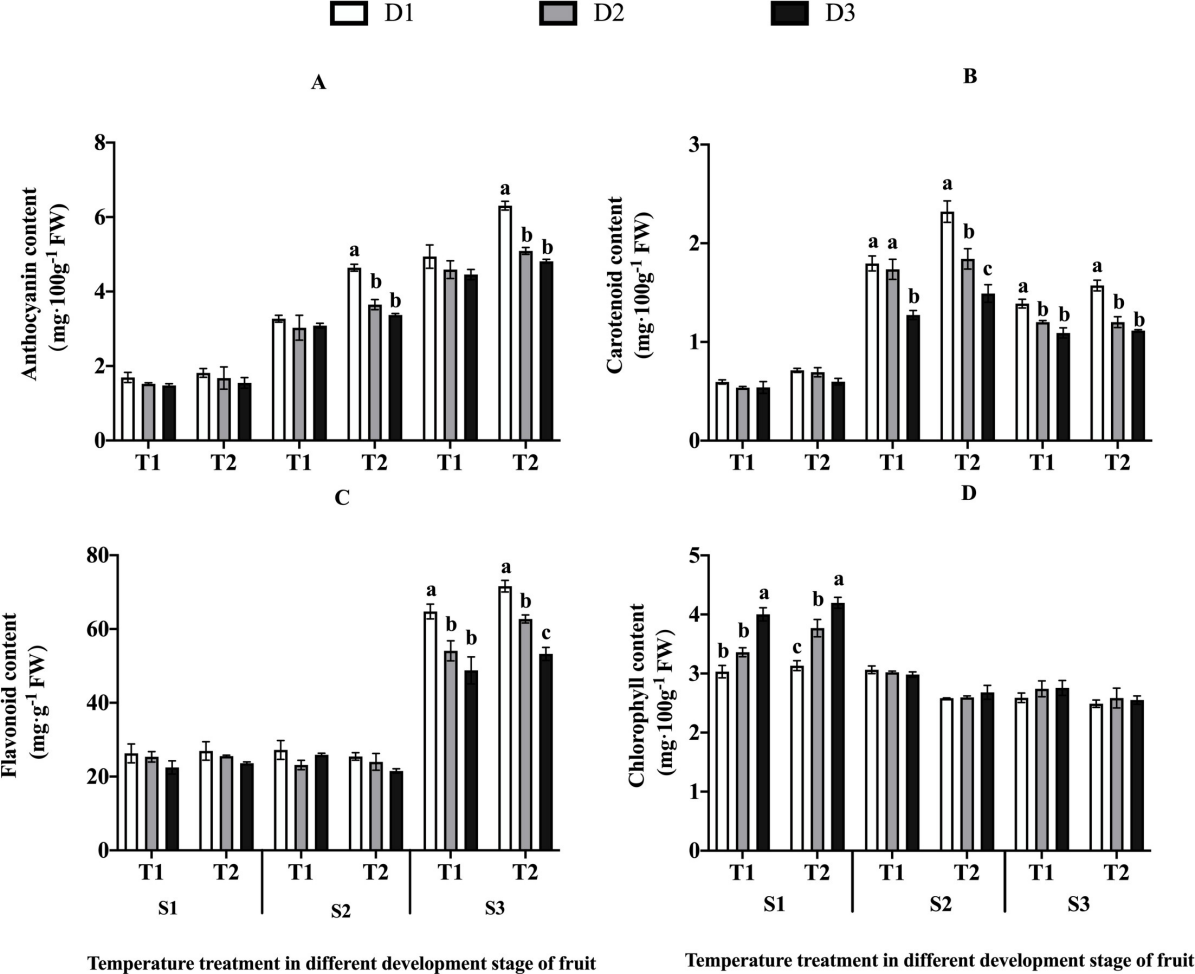
Pigments indices in jujubes. Values are means ± SE (n≥3). (A) The content of anthocyanin. (B) The content of carotenoid. (C) The content of flavonoid. (D) The content of chlorophyll. Different letters in bars indicate significant differences between means for the drought stress on different temperature treatments at the same period using Duncan’s test (*p*<0.05). * indicates elevated temperature has a significant effect on different drought stress.

In white ripening stage (S1), both temperature and drought stress showed significant (P<0.05) and highly significant (P<0.01) effects on chlorophyll content and only temperature had a highly significant affect (P<0.01) on the content of carotenoid (Table 3). Specially, the content of chlorophyll decreased under elevated temperature and increased when the drought stress increased (Fig 4D). The chlorophyll content increased more significantly with the intensification of drought stress (D2, D3) in T2 than that in T1, increasing by 20.28%, 33.94% compared with D1, respectively (Fig 4D).

**Table 3:** Results (*F* value) of two-way ANOVA on effects of elevated temperature and drought stress on the pigment content of peels.

		Anthocyanin content	Flavonoid content	Carotenoid content	Chlorophyll content
S1	Temperature	0.836	0.221	14.021**	7.499*
	Drought	1.236	2.186	2.847	46.803**
	Temperature × Drought	0.05	0.035	0.418	1.122
S2	Temperature	33.762**	1.905	14.933**	68.147**
	Drought	11.928**	1.951	28.871**	0.086
	Temperature × Drought	0.016	1.382	2.918	1.204
S3	Temperature	25.033**	12.645**	3.903	2.763
	Drought	16.517**	28.14**	42.181**	0.736
	Temperature × Drought	0.034	0.414	2.732	0.109

During fruit coloration stage (S2), both temperature and drought stress had highly significant effects on anthocyanin and carotenoid content and only temperature showed a highly significant effect on chlorophyll (Table 3). The content of anthocyanin and carotenoid increased under elevated temperatures and decreased with the intensification of drought stress (Fig 4A and 4B). Anthocyanin content decreased more significantly (P<0.05) with the intensification of drought stress under T2 group than that in T1, and increased more under T2 than that in T1 under the three drought stresses (D1, D2, D3) by 21.33%, 27.31% compared with D1, respectively (Fig 4B). Carotenoid content decreased more significantly (P<0.05) with the intensification of drought stress under T2 group than that in T1, by 20.62%, 19.12% compared with D1, respectively (Fig 4B).

During S3, there were highly significant effects on anthocyanin and flavonoid content among temperature and drought stress and only drought had a highly significant effect on the content of carotenoid (Table 3). Anthocyanin content decreased more significantly (P<0.05) with the intensification of drought stress under T2 group than that in T1, by 19.27%, 23.68% compared with D1, respectively (Fig 4A). Flavonoid content decreased more significantly (P<0.05) with the intensification of drought stress under T2 group than that in T1, by 12.41%, 25.63% compared with D1, respectively (Fig 4C). The content of carotenoid decreased more significantly (P<0.05) with the intensification of drought stress under T2 group than that in T1 (Fig 4B).

The changes in the content of pigments in different treatment combinations in the three developmental stages were also shown in Fig 4A–4C. The highest content of chlorophyll content was observed in S1 (T2D3), while the lowest content was in S3 (T2D1). For the content of carotenoid, the highest value was observed in S2 (T2D1) and the lowest was in S1 (T1D3). The highest value of flavonoid content was observed S3 (T2D1) and the lowest was in S2 (T2D3). For the content of anthocyanin, the highest content was in S3 (T2D1) and the lowest was in S1 (T1D3) (Fig 4).

### Enzyme activities

**Fig 5:**
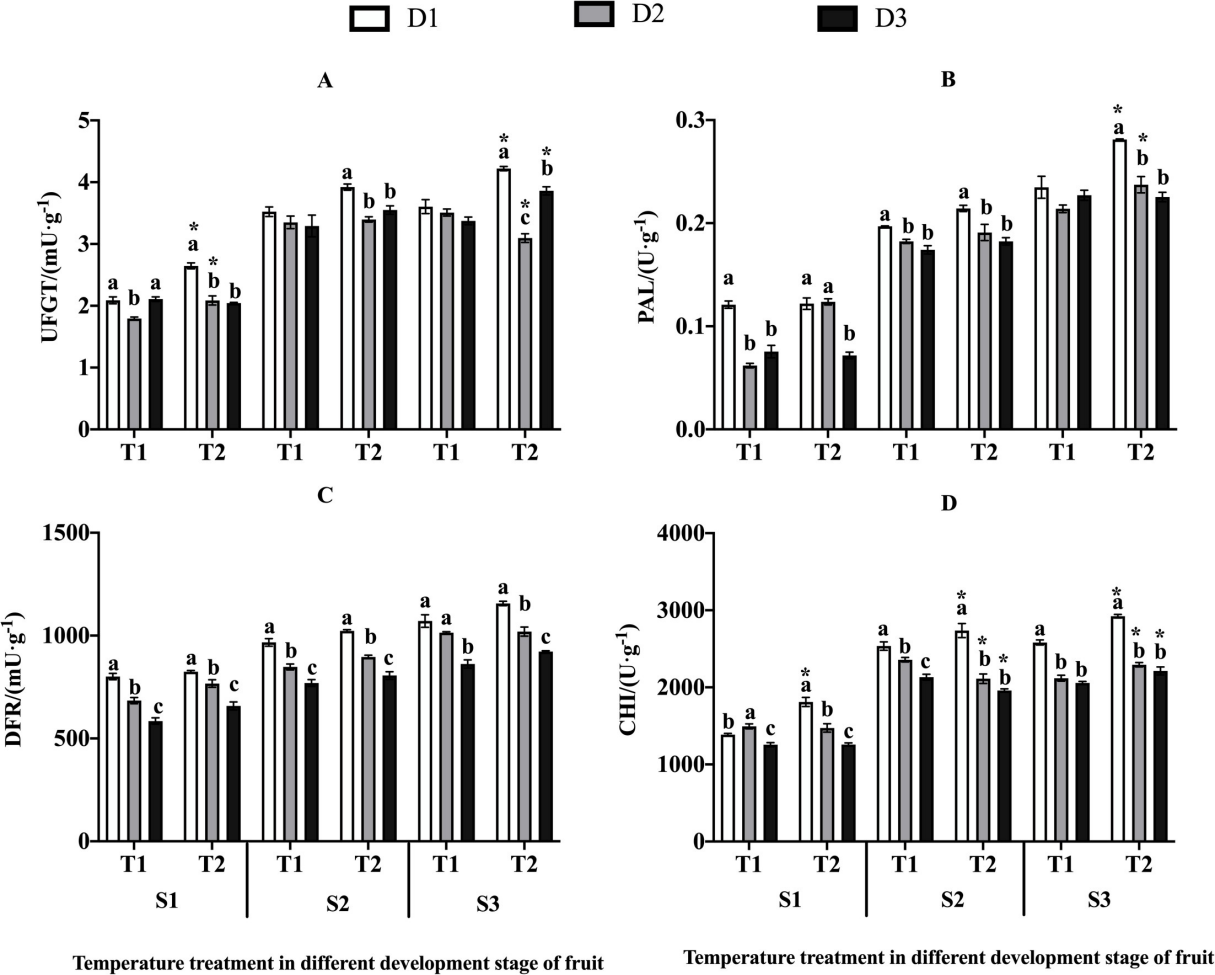
Enzymes indices in jujubes. Values are means ± SE (n≥3). (A) The activity of UFGT. (B) The activity of PAL. (C) The activity of DFR. (D) The activity of CHI. Different letters in bars indicate significant differences between means for the drought stress on different temperature treatments at the same period using Duncan’s test (*p*<0.05). * indicates elevated temperature has a significant effect on different drought stress.

For UFGT, both temperature and drought were highly significant (P<0.01) and affected the activity across S1 to S3, (Table 4). The activity of UFGT increased under elevated temperatures regardless the drought stress except in S3 (T2D2) and decreased as the drought stress intensified. The activity of UFGT decreased more significantly (P<0.05) as the drought stress intensified in T2 than that in T1 at S1, S2 and S3, and increased significantly (P<0.05) with elevated temperature under D1, D2 (S1) and under D1, D3 (S3), increasing by 26.68%, 16.36% and 17.08%, 14.42% respectively (Fig 5A). However, the activity decreased significantly when temperature increased under D2 in S3 (Table 4).

**Table 4:** Results of two-way ANOVA (F value) on effects of elevated temperature and drought stress on anthocyanin synthesis-related enzymes activity of peels.

		UFGT	PAL	DFR	CHI
S1	Temperature	47.388**	33.425**	21.773**	18.462**
	Drought	43.937**	66.644**	73.635**	40.893**
	Temperature × Drought	22.27**	38.373	2.067	21.194**
S2	Temperature	8.764*	11.426**	15.486**	2.653
	Drought	7.64**	22.843**	100.336**	61.237**
	Temperature × Drought	1.67	0.785	0.216	9.709**
S3	Temperature	15.775**	19.745**	11.492**	64.257**
	Drought	37.106**	17.491**	74.134**	195.283**
	Temperature × Drought	31.177**	7.381**	2.568	4.425*

For PAL, there was highly significant effects of elevated temperature and drought stress across S1 to S3, and significant Temp x Drought was found in S3 (Table 4). The activity decreased more significantly with the intensification of drought stress under T2 than that under T1 during S2 and S3, increased significantly with elevated temperature in D1, D2 during S3 by 15.58%, 19.83% (Fig 5B).

For DFR, both temperature and drought stress showed highly significant effects but no significant interaction between temperature and drought stress during three stages (S1, S2, S3) (Table 4). The activity of DFR increased significantly with elevated temperature and decreased significantly when the drought stress intensified regardless the temperature (Fig 5C).

Throughout all three stages (S1, S2, S3), the activity of CHI was significantly and highly significantly affected by the treatment of temperature and drought, as well as by their interaction (Temp. x Drought). The only exception was CHI activity in S2, where the elevated temperature showed no significant effect (Table 4). Specially, in S1, the activity of CHI decreased significantly as the drought stress intensified under T2 and increased significantly with elevated temperature in D1 (Fig 5D). CHI activity decreased significantly more when drought stress increased under T2 than that under T1 in S2 and S3. During S2, the activity increased significantly with elevated temperature in D1 but decreased in D2 and D3. And CHI activity increased significantly in T2 under three drought stress (D1-13.19%, D2-8.24%, D3-7.57%) (Fig 5D).

### Correlation among soluble sugar, organic acid, pigment and anthocyanin-related enzymes activity

The content of soluble sugar had highly significant positive correlations with the anthocyanin, carotenoid and flavonoid contents (R = 0.919 **, 0.377 **, 0.921 **), and had a highly significant negative correlation with the chlorophyll content (R = -0.688 **). For organic acids, there was a highly significant positive correlation with anthocyanin and carotenoid contents (R = 0.467 **, 0.800 **) and a highly significant negative correlation with chlorophyll content (R = -0.538 **). A highly significant correlation of anthocyanin synthesis-related enzymes was found with anthocyanin, carotenoid and flavonoid contents (Table 5).

**Table 5:** Correlation analysis among soluble sugar content, organic acid content, pigment content and anthocyanin-related enzymes activity of fruits under elevated temperature and drought stress.

	Soluble sugar	Organic acid	Anthocyanin	Carotenoids	Flavonoids	Chlorophyll	UFGT	PAL	DFR	CHI
Soluble sugar	1	0.290*	0.919**	0.377**	0.921**	-0.688**	0.715**	0.874**	0.796**	0.706**
Organic acid	0.290*	1	0.467**	0.800**	0.002	-0.538**	0.720**	0.581**	0.382**	0.649**
Anthocyanin	0.919**	0.467**	1	0.630**	0.794**	-0.796**	0.866**	0.945**	0.872**	0.850**
Carotenoids	0.377**	0.800**	0.630**	1	0.109	-0.640**	0.813**	0.690**	0.647**	0.829**
Flavonoids	0.921**	0.002	0.794**	0.109	1	-0.535**	0.509**	0.712**	0.730**	0.537**
Chlorophyll	-0.688**	-0.538**	-0.796**	-0.640**	-0.535**	1	-0.796**	-0.830**	-0.775**	-0.758**
UFGT	0.715**	0.720**	0.866**	0.813**	0.509**	-0.796**	1	0.905**	0.799**	0.901**
PAL	0.874**	0.581**	0.945**	0.690**	0.712**	-0.830**	0.905**	1	0.884**	0.884**
DFR	0.796**	0.382**	0.872**	0.647**	0.730**	-0.775**	0.799**	0.884**	1	0.879**
CHI	0.706**	0.649**	0.850**	0.829**	0.537**	-0.758**	0.901**	0.884**	0.879**	1

## Discussion

### The effect of elevated temperature on jujube quality attributes

Changes in fruit flavor and appearance critically influence quality of jujube fruit. The flavor of fresh eating jujube is largely determined by the content and ratio of soluble sugar and organic acid in fruits, whereas the fruit color resultant from changes in the content of chlorophyll, carotenoids, flavonoids and anthocyanins in the peel as the fruit matures [54–57]. In this process, the accumulation of fruit soluble sugar and peel anthocyanin are the important factors that determines the flavor and appearance color of the fruit in this process. Anthocyanins have anti-oxidant properties and anti-diseases to promote human health. The dark red fruits with sweet taste are more popular for consumers [58, 59]. Here we show that the elevated temperature (1.5–2.5° C than normal temperature) significantly increased the fruit sugar content, sugar-acid ratio, anthocyanins, flavonoids and carotenoids content. This result is compatible with previously reported results in assessing the effect of temperature on jujube., which reported that the soluble sugar and organic acid content of the jujube fruit increased, and the anthocyanin content increased by 0.16 mg·g-1 when the temperature increase of 2°C [60–62].

It is complex that the influence of growth environmental factors such as high temperature on fruit quality formation. Negative effects of high temperature on the quality of jujube and other fruits have also been reported. Doymaz [63] et al. found that high temperature caused poor coloring of jujube fruit. Some studies on anthocyanins showed that increasing temperature negatively affect anthocyanin content in different fruit crops including grapes [64, 65]; strawberries [66] and kiwifruit [67], typically due to the reduced anthocyanin synthesis and accumulation, decreased anthocyanin synthase activity, and decreased gene transcripts in the anthocyanin synthesis pathway. Our results, therefore, suggested that the effect of high temperature on the formation of fruit quality varied and depends on the growing environment of the fruit tree. Different varieties and growing environments have different optimal temperatures for fruit quality formation [68]. In the jujube producing area, the daytime temperature suitable for fruit growth and coloring is 24 to 30°C, while the nighttime temperature is 12 to 16°C [69]. Ningxia is located in northwestern China and is a temperate climate zone. Therefore, even if the temperature rises by 2°C, it is still within the normal range of jujube fruit development and coloration. The chlorophyll content in the fruit peel increases with increasing temperature, which may lead to an increase in photosynthetic products. Sugar is the main carbon source for anthocyanin synthesis, so the increase in temperature may indirectly increase the anthocyanin content by increasing the sugar content [70–72]. Yang [73] et al. treated the jujube plants in a similar manner to the simulated temperature increase, and the results showed that the sugar content in the jujube increased, which led to an increase in the synthesis of anthocyanins in the jujube variety ‘Lingwuchangzao’ [60]. The anthocyanin content has a significant positive correlation with soluble sugar content and the enzyme activities of PAL, CHI, UFGT, and DFR, with PAL having the highest correlation.

### The effect of moderate drought stress on jujube quality attributes

Moderate drought stress can improve the quality of fruits to a certain extent. The regulated deficit irrigation strategies (RDI) was used to improve the vegetative growth, yield, fruit quality and mineral nutrition of fruits [74, 75]. To improve fruit quality and anthocyanin content, growers have applied RDI to the production of wine grapes, apples, and tomatoes [76–80]. Several studies have shown that RDI has a positive effect on soluble solids and sugar accumulation in jujube [34, 35, 81]. It was suggested that RDI irrigation typically results in depressed plant growth, but may lead to photosynthetic products being distributed to the fruits, thus increasing the soluble solid content and sugar/acid ratio in jujube fruit [81, 82].

In the present study, we show that under the drought stress where the soil moisture is 30% -50% of the field capacity, sugar content, sugar/acid ratio of the fruit are significantly reduced when the drought stress increased. The negative impact on fruit quality differed from the results observed in the reported RDI experiments [34, 35, 81, 83], but was consistent with the reported studies carried out on the same cultivar in the same regions [60, 73]. In addition, the present study showed that drought stress decreased anthocyanin and carotenoid contents, as well as increasing the content of chlorophyll during fruit ripening, regardless of the atmospheric temperature (Table 3; Fig 4). The negative effect of drought treatment on jujube quality may be explained by the intensity of the drought treatment. In the studies that reported positive effect of RDI, soil water content ranged from 0.31 to 0.50 [34, 35, 81, 83], which is less severe than the current experiment (0.27). In addition to the intensity of drought treatment, timing, duration, and repetition of events of water deficit are also critical for the effect of drought on fruit quality, as reviewed by Ripoll [84] et al. In a semiarid area, such as Ningxia, soil moisture could have a more adverse effect on the fruit quality attributes, such as flavor and pigments.

### Enzyme activities and their correlation with quality attributes

The enzyme activities relating to anthocyanin biosynthesis were all significantly affected by elevated temperature and drought treatments. Anthocyanin biosynthesis in plants belongs to a branch of the flavonoid metabolism pathway. The process can be divided into three stages and is catalyzed by different enzymes [85–87]. PAL is an important enzyme that catalyzes phenylalanine to cinnamic acid in the first stage. CHI and DFR play important role in the second stage of anthocyanin biosynthesis. Together with F3H and CHS, CHI and DFR catalyze the production of dihydroflavonols using the products of the first stage in anthocyanin biosynthesis. In the last stage, Anthocyanin synthase/leucocyanidin dioxygenase (ANS/LDOX) catalyzes the formation of anthocyanidins. The formed anthocyanidins are unstable at this time and must undergo a series of methylation, glycosylation, and acylation reactions to form stable anthocyanins. The stabilized anthocyanins are then transported by UDP glucose-flavonoid-3-O-glycosyltranferase (UFGT). This stage is the key metabolic pathway for anthocyanin exhibit color in plants [88].

Result of the present study showed that the elevated temperature (1.5–2.5°C than normal temperature) enhanced enzymes activities such as PAL, CHI, DFR, and UFGT, which are closely related to the synthesis and accumulation of anthocyanins. The drought treatment, however, decreased the activity of these enzymes. The trend of these changes is compatible with the pattern of anthocyanin content (Fig 5; Table 4). In addition, highly significant positive correlation was observed between soluble sugar content and the activities of PAL, CHI, UFGT and DFR. These correlations appeared consistent with previously reported studies in other fruits. For example, PAL activity has been reported to positively correlate with anthocyanin synthesis in grapes [89], strawberries [90] and apples [21]. Correlation between CHI activity and anthocyanin synthesis was reported in apples [91] and pear [92]. Ju [93] et al. found that DFR activity was higher in the red fruited apple cultivars than that in non-red fruit cultivars. Moreover, the increased activity of UFGT found in this study is in agreement with the recent finding based on transcriptome analysis [94], which showed that UFGT genes involved in the accumulation of anthocyanins were significantly increased in the last ripening periods of jujube fruits. They further suggested that reducing the activity of UFGT genes during jujube ripening could be a potential approach to maintain good fruit appeal under long-term storage [94]. The current results indicate that jujube growers must consider not only the influence of temperature, but also the severity of drought stress on jujube coloration, which depends to a large extent on the metabolism of anthocyanins [95].

### Implication on production of jujube “LingwuChangzhao” in Ningxia, China

Ningxia is in the arid and semi-arid areas of northwest China, with a highly fragile ecological environment. The annual average precipitation is less than 500 mm and studies have shown that the evaporation in northwestern China and drought levels will increase due to climate warming [50, 96, 97]. Global climate change is affecting and will continue to affect ecosystems worldwide. Specifically, temperature and precipitation are both expected to shift globally, and their separate and interactive effects will likely affect ecosystems differentially depending on current temperature, precipitation regimes, and other biotic and environmental factors. The present results show that the fruit quality of jujube variety "Lingwuchangzao" can be improved when the atmospheric temperature increases by about 2° C in this region. However, drought stress, at less than 50% of the field water holding capacity, has a negative impact on the fruit's sugar-acid ratio and pigment content. The present results also showed that the synthesis and accumulation of anthocyanins in jujube fruit were positively correlated with sugar content and related enzyme activities, especially Phenylalanine Ammonia-lyase (PAL) activity. This study is an important step in understanding how environmental factors, associated with climate changes, may impact jujube industry in Ningxia, China. Under the background of global warming, jujube farmers in Ningxia should pay more attention to the impact of drought stress on the quality of jujube and mitigate the adverse effect. This study, provides novel information for understanding the influence of growth environment on the quality properties of jujube fruits. This knowledge will help develop appropriate crop management practices for jujube production in arid and semi-arid areas in northwest China.

## Conclusions

In this study, we evaluated and analyzed the soluble sugar, organic acid content, the pigment content and the activity of the enzymes related to anthocyanin of the jujube cultivar ‘Lingwuchangzao’ with different stages of fruit maturation. The results showed that the fruit sugar content, sugar-acid ratio, anthocyanins, flavonoids and carotenoids content were significantly increased by the elevated temperature (1.5–2.5° C than normal temperature). Under the same temperature, the sugar content, anthocyanin, flavonoid and carotenoid content of the fruit were significantly reduced by the drought stress especially when the soil moisture was 30%-50% of the field capacity, but the chlorophyll and organic acid content increased. The current results show that when the atmospheric temperature in the region rises by about 2°C, the fruit quality of the jujube variety "Lingwuchangzao" will be improved. However, drought stress has a negative impact on the sugar-acid ratio and pigment content of the fruit. The results of this study also show that the synthesis and accumulation of anthocyanins in jujube fruits are positively correlated with sugar content and related enzyme activities, especially phenylalanine aminolyase (PAL) activity. Therefore, this study provides novel information for understanding the influence of growth environment on the quality characteristics of jujube fruit. This knowledge will help develop appropriate crop management practices for jujube production in the arid and semi-arid regions of Northwest China.

## Supporting information

S1 Table Relevant data underlying the findings described in manuscript. (XLSX)
